# Longitudinal MRI-based changes in intracranial volume and skull thickness observed in both metachromatic leukodystrophy and multiple sclerosis

**DOI:** 10.1016/j.nicl.2026.103968

**Published:** 2026-02-16

**Authors:** Guus H.J. Vorst, Nicole I. Wolf, David R. van Nederpelt, Frederik Barkhof, Marjo S. van der Knaap, Menno M. Schoonheim, Eva M.M. Strijbis, Petra J.W. Pouwels

**Affiliations:** a Amsterdam Leukodystrophy Centre, Department of Child Neurology, Emma Children’s Hospital, and Amsterdam Neuroscience, Amsterdam University Medical Center, Vrije Universiteit, Amsterdam 1105 AZ, the Netherlands; b MS Center Amsterdam, Department of Radiology and Nuclear Medicine, Amsterdam Neuroscience, Amsterdam University Medical Center, Amsterdam 1081 HV, the Netherlands; c UCL Queen Square Institute of Neurology and Centre for Medical Image Computing, University College London, London WC1N 3BG, United Kingdom; d MS Center Amsterdam, Department of Anatomy & Neurosciences, Amsterdam Neuroscience, Amsterdam University Medical Center, Amsterdam 1081 HV, the Netherlands; e MS Center Amsterdam, Department of Neurology, Amsterdam University Medical Center, Amsterdam 1081 HV, the Netherlands

**Keywords:** Skull, Intracranial volume, Neurodegeneration, Multiple sclerosis, Metachromatic leukodystrophy

## Abstract

Intracranial volume (ICV) is often used as normalization factor in volumetrics and considered to be stable in young adults. We noticed thick skulls on MRI scans of leukodystrophy patients, suggesting potentially changing skull morphology. In this study we aimed to quantify skull thickness and ICV in metachromatic leukodystrophy (MLD) patients and people with multiple sclerosis (pwMS). We retrospectively analyzed single-center cross-sectional and longitudinal MRI scans. Skull thickness and ICV were determined using automated segmentation techniques. Participants included MLD (n = 32, 11 male, scans = 136, median age first scan = 14.1 [IQR 7.9–25.7] years), MS (n = 232, 78 male, median age first scan = 47.3 [IQR 39.6–55.4] years, scans = 431), and controls (n = 139, 67 male, median age first scan = 30.7 [IQR 10.7–48.9] years, scans = 283). Both ICV and skull thickness showed natural growth in young controls. In young MLD participants, ICV decreased (−18.8 ± 22.4 mL/year, *p* < 0.001). Above age 20, ICV and skull thickness remained stable in controls. In comparison to controls, we observed ICV loss in MLD participants (−4.01 ± 8.29 mL/year, *p* < 0.001) and in pwMS (−2.99 ± 2.69 mL/year, *p* < 0.001), as well as skull thickening (MLD: 0.16 ± 0.14 mm/year, *p* < 0.001, pwMS: 0.04 ± 0.09 mm/year, *p* = 0.009). In adult patient groups, negative correlations were found between ICV and skull thickness (MLD: −19.24 mL/mm, *p* < 0.001, pwMS: −11.56 mL/mm, *p* < 0.001), but not in controls (*p =* 0.11). Despite limitations due to scanner variations, segmentation reliability and absence of validation against ground truth, these findings demonstrate a reduction in ICV in pathologies, already in young adulthood. Although the observed changes are small, they may lead to underestimations of atrophy when using ICV as a normalization factor.

## Highlights

• ICV decreases over time in patients with metachromatic leukodystrophy and people with MS.
• Skull thickness increases over time in patients with metachromatic leukodystrophy and people with MS.
• Increased skull thickness and decreased ICV are inversely related in MLD and MS, but not in controls.
• Skull morphology is not stable and may adapt to brain atrophy.
• Using normalized volumes may underestimate atrophy.

## Graphical abstract

**Figure:**
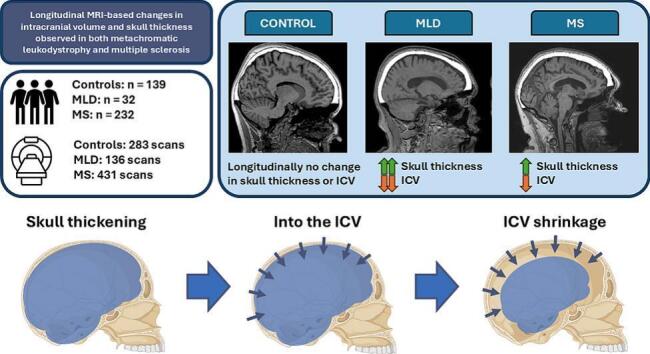

## 1 Introduction

Volumetric assessment of brain tissue is widely used to study morphological differences between subjects in a large variety of brain disorders, such as dementia, multiple sclerosis (MS), but also in leukodystrophies (Tillema et al., 2015, Al-Saady et al., 2024, Chard et al., 2004, Miller et al., 2002, Scahill et al., 2003, Schippling et al., 2017, Buckner et al., 2004). Leukodystrophies are rare, genetic disorders affecting mainly the white matter, sometimes leading to atrophy already at a young age. To account for variability in head size between subjects, brain volumes are often expressed as normalized volumes, using intracranial volume (ICV) as a normalization factor (Voevodskaya et al., 2014). During qualitative assessment of conventional MRI scans in people with leukodystrophies, we regularly observed remarkably thick skulls. This observation prompted us to perform a systematic quantitative assessment of skull thickness. Given that changes in skull morphology may influence the ICV, in this study we also focus on ICV (Royle et al., 2013, Nerland et al., 2022).

ICV increases during development (Sgouros et al., 1999), but it is generally assumed to remain constant after reaching adulthood (Voevodskaya et al., 2014). This has been demonstrated in cross-sectional observations in healthy subjects (Courchesne et al., 2000, Schippling et al., 2017) and dementia patients (Buckner et al., 2004). Differences in ICV between different age-groups have been interpreted as being linked to secular trends of increasing head size over the past century, rather than effects of within-subject longitudinal change (Good et al., 2001, Decarli et al., 2024). These studies were based on cross-sectional data, which may not fully capture true individual trajectories of age-related or disease-specific changes in ICV. Using both a longitudinal and cross-sectional approach Caspi *et al.* (Caspi et al., 2020) observed that ICV shows a small increase during young adulthood and a slight decrease from the fourth decade of life in a healthy population. A study by Nerland *et al.* showed that cross-sectional ICV estimations were not consistent, since both positive and negative associations with age were observed, depending on segmentation method and investigated dataset (Nerland et al., 2022). However, longitudinal analyses showed strong evidence for a small ICV reduction in mid- to late adulthood in a healthy population (Nerland et al., 2022). As intracranial volume is constrained by the inner skull border, a longitudinal ICV reduction may partly reflect age-related changes in skull morphology. A manual segmentation study that explicitly accounts for inner skull thickening has shown measurable decreases in ICV in older adults (Royle et al., 2013). However, longitudinal MRI data assessing ICV and skull thickness in neurological disease populations remain limited.

The current study aimed to quantify skull thickness and ICV both cross-sectionally and longitudinally. We included control subjects and two types of pathology: metachromatic leukodystrophy (MLD) and MS. MLD was selected as a leukodystrophy, as it includes early-onset atrophy and is relatively frequent among leukodystrophies (Al-Saady et al., 2024, Asbreuk et al., 2025). MS was included to investigate whether possible changes in skull thickness and ICV could occur in a common, non-genetic neuroinflammatory disease with progressive brain atrophy (Chard et al., 2004, Miller et al., 2002). Together these groups allowed us to evaluate the potential effect of distinct brain disorders on skull thickness and the ICV.

The primary objective was to determine whether ICV and skull thickness change over time in patients with MLD, people with MS (pwMS), and controls. The secondary objective was to evaluate the association between skull thickness and ICV in these groups. A third objective was to assess whether skull-thickening was a global diffuse phenomenon, or whether there were regional differences in skull thickening.

## 2 Materials and methods

### 2.1 Data acquisition

This retrospective study was conducted using data from cohorts, previously approved by the institutional Medical Research Ethics Committee. All participants, or parents/guardians, gave written informed consent according to the Declaration of Helsinki.

We retrospectively analyzed cross-sectional and longitudinal MRI scans of participants with MLD (*n* = 32, scans = 136), MS (*n* = 232, scans = 431), and controls (*n* = 139, scans = 283), giving a total of 403 subjects, with 850 MRI scans. Scans from MLD subjects were acquired from the department of child neurology at Amsterdam UMC, as described previously (Al-Saady et al., 2024, Tillema et al., 2015). Diagnosis was confirmed through both genetic and metabolic testing, which identified biallelic pathogenic *ARSA* variants in the gene, low arylsulfatase A activity and elevated urine sulfatide levels, following current practice (Thakkar et al., 2024, Asbreuk et al., 2025). MRI scans from pwMS were obtained from the Amsterdam MS Cohort, as described in previous studies (Eijlers et al., 2017, Eijlers et al., 2018, Meijer et al., 2017, van Nederpelt et al., 2025). This cohort includes both healthy controls and participants with clinically definite MS, as defined by Polman *et al.* (Polman et al., 2011). Control subjects were either healthy controls included from the Amsterdam MS Cohort, or patients with mild neurological complaints without MRI abnormalities. In the latter subset, some participants had a genetic diagnosis of X-linked adrenoleukodystrophy (X-ALD) under routine surveillance, who were neurologically normal and had normal MRI’s, and guanidinoacetate methyltransferase (GAMT) under creatine supplementation from birth. In both conditions, MRI can remain structurally normal for extended periods in clinically stable individuals (Engelen et al., 2012, Mercimek-Andrews and Salomons, 1993). Thus, these scans were considered suitable as control data to allow access to longitudinal datasets with repeated MRI acquisitions, which are otherwise difficult to obtain in healthy pediatric controls. For the MLD cohort and the control subjects in the younger age range, data have been acquired between 2000 and 2024 on 1.5 T (Siemens Vision, Siemens Sonata, Siemens Avanto) or 3 T (GE Discovery MR750, Philips Ingenia) MR scanners. Children younger than 1 year old were excluded from the study, as segmentation techniques for MRI data are less accurate in this age group. For the Amsterdam MS cohort, data have been acquired on the same 3 T scanner, although with a hardware upgrade between baseline (GE Signa HDxt) and follow-up (GE Discovery MR 750). In all cases, 3D-T1-weighted (3DT1w) images were used, and T2-weighted (T2w) scans if available.

### 2.2 Data processing

**Fig. 1:**
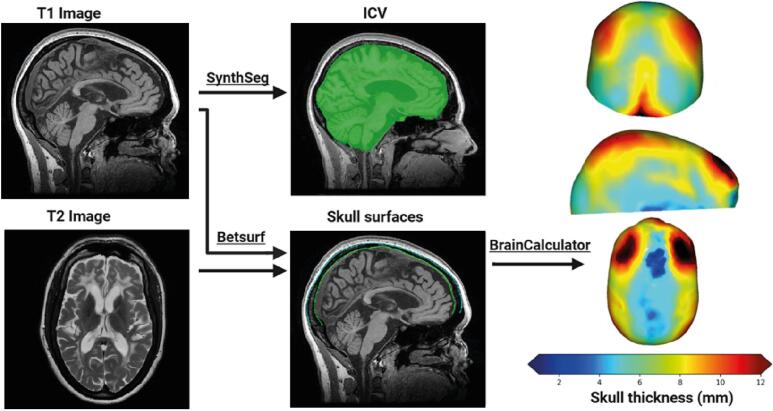
MRI Processing Pipeline: ICV was calculated using SynthSeg on 3D T1w images. Meshes of the inner and outer skull surfaces were extracted with Betsurf from T1w images (and T2w images if available). BrainCalculator generated point clouds from these meshes to compute skull thickness by calculating the Euclidean distance between the nearest points on each surface. The regional variation in skull thickness is shown in frontal, lateral, and top views.

A flow-chart of the analysis is shown in Fig. 1. ICV was obtained from 3DT1w images using Synthseg, as this contrast-agnostic segmentation algorithm is unaffected by white matter hypointensities on T1w images (Billot et al., 2023). After N4-bias field correction and brain extraction using HD-BET, the inner and outer skull surface meshes were determined using Betsurf from FSL (Smith, 2002, Isensee et al., 2019). T2w images with full brain coverage were used whenever available, because the high signal intensity of CSF on these images increased the segmentation accuracy (Jenkinson et al., 2005). T2w scans were not available for the Amsterdam MS cohort. Skull surface segmentations were visually inspected to check for accurate segmentation. To illustrate this quality control and the impact of using either T1w-only or T1w and T2w inputs, examples of both accepted and rejected segmentations are shown in Supplementary Fig. 1**.** In most cases, usage of both T1w and T2w images yielded a reliable segmentation of the skull. Cases in which skull segmentation produced unreliable meshes were excluded from further analyses. Following visual quality control, subsequent analyses were performed on the accepted meshes, regardless of whether they were derived from T1w-only or T1w + T2w input. The distribution of T1w-only and T1w + T2w input can be seen in Table 1.

**Table 1:** Demographic information and MRI measurements extracted per group.

	**Group**		
	**MLD**	**MS**	**Controls**
**Participants, n (% of total)**	**32 (7.9)**	**232 (57.6)**	**139 (34.5)**
Sex, Male (% of total)	11 (34.4)	78 (33.6)	67 (48.2)
Age at first scan (y, *Median* [IQR])	14.1 [7.9–25.7]	47.3 [39.6–55.4]	30.7 [10.7–48.9]
**Subjects with only cross-sectional scans**	**0**	**33**	**77**
**Subjects with longitudinal scans < 20y**	**20**	**0**	**12**
**Subjects with longitudinal scans > 20y**^a^	**14**	**199**	**52**
ICV Measurements	136	431	283
Skull thickness values	117	246	214
From T1w & T2w	90	0	67
From T1w only	27	246	147

Abbreviations: MLD = metachromatic leukodystrophy, MS = multiple sclerosis, IQR = Interquartile range, ICV = intracranial volume.

a a few MLD participants and controls had at least 2 longitudinal scans before the age of 20 years, and above the age of 20 years, the reason why the sum of cross-sectional, longitudinal < 20y and longitudinal > 20y exceeds the number of participants.

For further analysis, we removed the skull base and orbitofrontal regions using an exclusion mask in Montreal Neurological Institute (MNI) standard space and considered only (supratentorial) brain and skull superior from an axial slice at *z* = 28 (in MNI-2 mm) (Supplementary Fig. 2). Next, 5.0·10^5^ points were uniformly sampled on both skull surface meshes to generate point clouds using BrainCalculator (Zhang et al., 2023). For each outer skull point, the distance to the inner skull was calculated as the average distance of the 30 nearest neighbours at the inner skull to eliminate outliers in point sampling, with bounds applied to limit unreasonable values (0.5–12 mm) (Zhang et al., 2023). For each scan, we calculated the median skull thickness because thickness was typically non-normally distributed, as illustrated on the coloured skull thickness maps in Fig. 1.

To assess possible regional differences in skull thickness, we segmented the outer skull surface mesh into frontal, occipital, parietal, and temporal bones. These four regions were extracted from the calvarial bone atlas in MNI space (Kalc et al., 2024). We dilated this atlas with an 8 mm Gaussian kernel to ensure full overlap with the outer skull surface mesh (Supplementary Fig. 2). We registered this dilated atlas to subject space and classified the 5.0·10^5^ points into the four skull regions, based on their relative surface. Finally, using BrainCalculator, we calculated median thickness for each skull region.

### 2.3 Statistical analyses

Statistical analyses were performed using RStudio (RStudio Team, version 4.3.2). We visually inspected scatterplots of ICV and skull thickness as a function of age to assess overall trends over time. Here, we stratified the data by sex as it has been shown that males generally have a larger ICV, while females tend to have thicker skulls (Eliot et al., 2021, Eksi et al., 2021). Then, the dataset was split into ages above and below 20 years to account for natural skull growth until 20 years of age (Caspi et al., 2020, Almuhawas et al., 2020). The younger group only included MLD and control measurements, as all pwMS were older than 20 years. For both age groups separately, linear regression models were fitted to calculate slopes (Δ) in ICV and skull thickness per year for each subject. Although absolute values of ICV and skull thickness may differ between males and females, we had no indication that the slopes of the respective changes depend on sex. Therefore, we combined sexes for longitudinal analysis. Normality of data was assessed through the Shapiro-Wilk test or Kolmogorov-Smirnov test, depending on sample size, and Levene’s test was used to test for equal variance. For the younger two groups (MLD and controls), slopes were compared with t-tests. The older three groups (MLD, pwMS and controls) were compared with Kruskal-Wallis, as none of these groups met the assumptions of normality and homogeneity of variance. If applicable, Dunn’s test with Bonferroni correction was used for post-hoc pairwise comparisons. For skull subsegments, a similar statistical approach was used within each group to test whether potential skull thickness changes were region-specific or reflected a global pattern. Mixed-effects linear regression models were performed to evaluate the potential relation between ICV and skull thickness, with ICV as the dependent variable, skull thickness and sex as the fixed effects, and subject as a random intercept to account for individual variability (using the lme4 package version 1.1–35.3). For all statistical tests *p* < 0.05 was considered statistically significant.

## 3 Results

### 3.1 Demographics

Distribution of scans, demographic characteristics, and scan-related statistics of subject populations are summarized in Table 1. All data could be included for ICV, but some of the measurements were excluded for skull thickness determinations, after visual quality check. This was the case for 14% of MLD, 43% of pwMS, and 24% of control scans. Included skull segmentations were based on both T1w and T2w scans in 77% of MLD, 0% of pwMS, and 31% of control datasets, remaining segmentations were based on T1w scans only.

### 3.2 Visual assessment of ICV and skull thickness as function of age

**Fig. 2:**
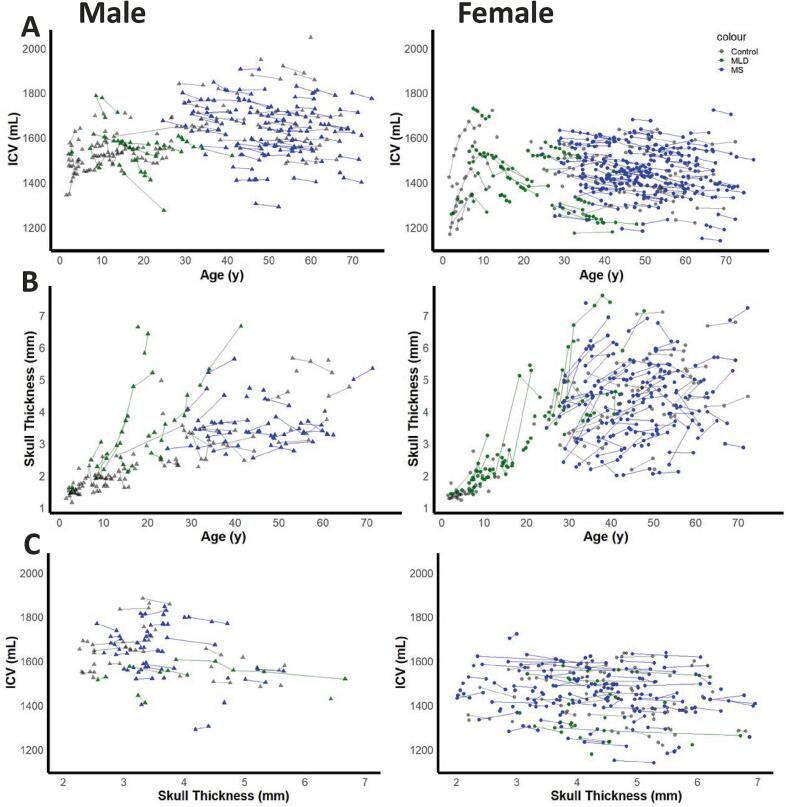
Cross-sectional and longitudinal changes in ICV and skull thickness over time. Data is represented as a function of age in males (left) and females (right) for (a) ICV and (b) skull thickness. (c) The relationship between skull thickness and ICV by sex and disease group, only including scans taken at an age above 20 years. Each line represents a subject’s measurements over time. Each point represents an individual measurement with circles for females and triangles for males. Colours indicate Control (grey), MLD, (green), and MS (blue). (For interpretation of the references to colour in this figure legend, the reader is referred to the web version of this article.)

Fig. 2 shows scatterplots of ICV and skull thickness as function of age. In controls, ICV increases during childhood and adolescence, and shows a plateau in adults. ICV is highly variable between subjects and becomes larger in males than in females (Fig. 2A). Skull thickness also increases in controls and becomes thicker in females than in males (Fig. 2B). In subjects with MLD a decrease of ICV and a steep increase in skull thickness are seen, for some individuals already around the age of 10 years (Fig. 2A, 2B). Several pwMS show a similar but more subtle pattern for ICV and skull thickness.

To visualize the possible influence of scanner, we have illustrated the longitudinal values for ICV and skull thickness for four control subjects and four MLD subjects, with multiple longitudinal scans (6–16 scans per subject), stratified by scanner, in Supplementary Fig. 3. From these plots, we noticed some systematic influence of scanner on both skull thickness and ICV. However, the magnitude of variation is small compared to the disease-related effects (please note that the graphs in Fig. 2 and Supplementary Fig. 3 have the same y-axis scaling).

### 3.3 Young age group (below 20 years)

**Fig. 3:**
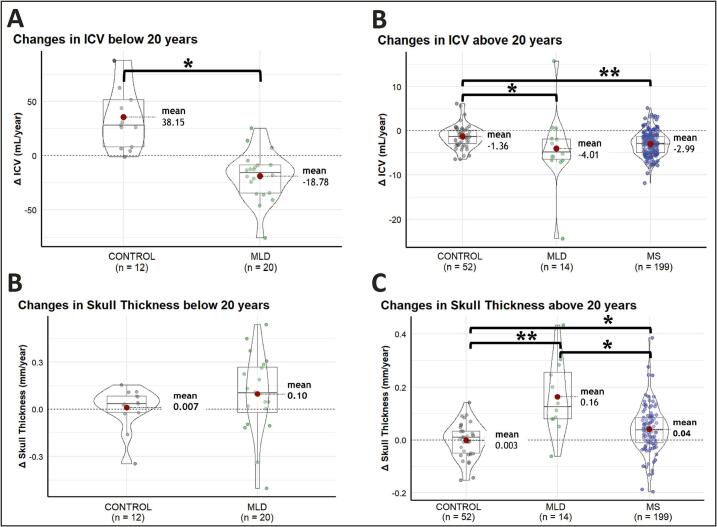
Violin plots of within-subject changes per year in ICV and skull thickness. Changes per year in ICV (a,b) and skull thickness (c,d). Panels (a) and (c) show subjects below 20 years (Control, MLD), while panels (b) and (d) show subjects above 20 years (Control, MLD, MS). Each point represents an individual subject’s change. Controls are shown in grey, MLD in green and MS in blue. * *p* < 0.05, ** *p* < 0.001. (For interpretation of the references to colour in this figure legend, the reader is referred to the web version of this article.)

Slopes in ICV and skull thickness below the age of 20 years are shown in Fig. 3A and 3C. The change in ICV in MLD participants was negative (ΔICV = −18.8 ± 22.4 mL/year), which was significantly different compared to the natural growth in controls (ΔICV = 38.2 ± 29.5 mL/year, *p* < 0.001). Although a few MLD participants had increases in skull thickness > 0.3 mm/year, mean Δskull thickness was not significantly different between MLD (Δ = 0.10 ± 0.25 mm/year) and controls (Δ = 0.007 ± 0.14 mm/year, *p* = 0.21).

### 3.4 Older age group (above 20 years)

Slopes in ICV and skull thickness above the age of 20 years are shown in Fig. 3B and 3D. The change in ICV was significantly different across all groups (*p* < 0.001). Post-hoc analyses revealed the biggest change in MLD participants (ΔICV = −4.01 ± 8.29 mL/year), compared to controls (ΔICV = −1.36 ± 2.51 mL/year, *p* < 0.001). Significant differences were also seen between pwMS (ΔICV = −2.99 ± 2.69 mL/year) and controls (*p* < 0.001). No difference was observed between MLD and pwMS (*p* = 0.44).

Mean Δskull thickness was significantly different across all groups (*p* < 0.001). Post-hoc analyses revealed that the observed change was higher in MLD participants (Δ = 0.16 ± 0.14 mm/year) than both pwMS (Δ = 0.04 ± 0.09 mm/year, *p* = 0.003) and controls (Δ = -0.003 ± 0.064 mm/year, *p* < 0.001). The increase of skull thickness was also significantly larger in pwMS than in controls (*p* = 0.009).

### 3.5 Association between skull thickness and ICV

Scatterplots of ICV versus skull thickness in the age range above 20 years are shown in Fig. 2C. A mixed-effects regression model revealed a significant negative association between ICV and skull thickness in MLD (*β* = -19.24 mL/mm, *p* < 0.001) and in MS (*β* = -11.56 mL/mm, *p* < 0.001), after adjusting for sex. There was no significant association between ICV and skull thickness in controls after adjusting for sex (*β* = -9.91 mL/mm, *p* = 0.11).

### 3.6 Regional skull thickness

We only considered frontal, parietal, and occipital skull bones for a regional analysis, because the relative surface of the temporal bones in the selected mask is very small, in some subjects less than 1%. For visualisation, we showed the mean regional skull thickness over all measurements for females and males above 20 years in Supplementary Fig. 4. Generally, the frontal skull is thickest, especially in females. Longitudinal analysis of regional skull thickness revealed no significant differences in the rate of change between cranial regions in any group. The pattern of change was similar across regions for subjects with MLD (*p* = 0.21), pwMS (*p* = 0.22), and controls (*p* = 0.52), indicating that skull thickness changes were global rather than region-specific.

## 4 Discussion

In this study, we quantified skull thickness and the possible relationship with ICV. In control subjects the skull thickened during childhood and adolescence and remained relatively constant in our adult group. We observed skull thickening in pwMS and especially in the MLD group. In controls, ICV increased during development and remained relatively constant during adulthood. In contrast, ICV decreased in pwMS, and especially in MLD, already during adolescence for some patients. In adults with MS and MLD, skull thickness was negatively associated with ICV. This association was not present for adult controls. Skull thickening and increase in ICV during childhood and adolescence, as observed in the control subjects, belong to normal development (Sgouros et al., 1999). In several subjects with MLD, we noticed more severe skull thickening already during adolescence, and while ICV continued to increase in control children and adolescents, it decreased in the young MLD group. This pattern of skull thickening and ICV shrinkage continued in the adult MLD group. In adult pwMS, we observed a similar pattern, although less pronounced.

The observations in our longitudinal study regarding control subjects agree with findings of stable ICV during adulthood in previous cross-sectional studies (Courchesne et al., 2000, Schippling et al., 2017, Buckner et al., 2004). Our control group did not exhibit significant ICV shrinkage or skull thickening, which may be due to the younger age range and short follow-up time in this study, compared to other longitudinal studies which reported small within-subject ICV changes within an older population (Royle et al., 2013, Caspi et al., 2020, Nerland et al., 2022).

Since the present findings in our MLD and MS group are based on longitudinal within-subject changes with generation-matched controls, we presume that an active biological response may play a role in ICV reduction and skull thickening in these pathologies. Royle *et al.* demonstrated a relationship between ICV and skull thickness, finding inner table skull thickening and ICV shrinkage in healthy adults in their seventies, leading to potential underestimations of brain atrophy (Royle et al., 2013). This supports the hypothesis that skull thickening into the intracranial space is an active mechanism, particularly in the context of neurodegeneration, considering that age-associated atrophy is accelerated after 70 years in healthy individuals (Scahill et al., 2003). At older age (particularly in postmenopausal women), hyperostosis frontalis interna, a benign thickening of the frontal bone, may also occur (Raikos et al., 2011, Devriendt et al., 2005). In our cohorts we found no regional difference in skull thickening to support this, but these studies show that the morphology of the skull can change in adults.. Skull thickening and ICV shrinkage may be linked to pathological processes. We observed these phenomena in both MLD, a genetic disorder, and MS, an acquired disorder. Although underlying disease mechanisms differ, both diseases affect white matter, but neurodegeneration and atrophy occur as well (Al-Saady et al., 2024, Miller et al., 2002). Consistent with this pathological context, a study by Mortazavi *et al.* found that MS diagnosis at an earlier age was strongly associated with a lower ICV (Mortazavi et al., 2024). Together with our findings, this supports the notion that neurological disease mechanisms can influence intracranial volume.

Results from our study cannot explain the cause of skull thickening and ICV shrinkage, nor whether these phenomena are causally related, but atrophy and ensuing reduced intracranial pressure may play a role. A relationship between atrophy and intracranial pressure has been suggested in numerical models in pwMS (Abdi et al., 2023). Computational biomechanical investigations further support the idea that brain tissue integrity as well as changes in bone density can modify intracranial pressure (Abdi et al., 2024). Supporting this, studies have demonstrated disease-related alterations in mechanical properties of the brain and intracranial tissue, when conventional volumetrics appear relatively preserved (Abdi et al., 2025, Streitberger et al., 2012). Skull thickening has also been observed in subjects with ventricular drainage as treatment of hydrocephalus, and atrophy in adults after traumatic brain injury or neurodegenerative disorders (Wolf and Falsetti, 2001, Kaufman et al., 1973). A review by Benson *et al.* highlighted that the calvarial bone is capable of dynamic structural changes in response to altered intracranial pressure (Benson et al., 2023). Low intracranial pressure may lead to the skull growing inward along its inner table, yielding a thicker skull and a concomitant reduction in ICV, while high pressure is associated with expansion (Benson et al., 2023). Taken together, these observations provide a potential mechanistic framework in which disease-related brain atrophy and altered tissue mechanics lead to reduced intracranial pressure, triggering compensatory skull remodeling that reduces the ICV.

Importantly, this hypothesis is supported by indirect evidence and further research is needed to confirm and explain the effects we see in this study. Similar phenomena may occur in other neurodegenerative diseases, but generalization should be made with caution. While our results are statistically significant, the observed changes are small. ICV shrinkage may not have a direct pathological consequence for patients, but it can confound volumetric measurements of e.g. brain atrophy, when ICV is used as a normalization factor. Prior studies have shown that different normalization methods can meaningfully influence the relationships between brain volumes, ICV, and other outcome measurements (Voevodskaya et al., 2014, Wang et al., 2024). Therefore, it is important to account for potential ICV changes when interpreting normalized brain volumes in neurodegenerative studies. Subtle ICV shrinkage may possibly serve as a complementary marker for detecting disease-related changes beyond standard volumetrics and be used to characterize disease progression and study the effects of emerging therapies (Medina et al., 2024).

A limitation of this study is the use of data from different scanners and field strengths, which is inevitable in a longitudinal study over such a long time, and in a disease with a low prevalence, such as MLD. In fact, the long follow-up time and relatively large number of MLD participants should be considered a strength of this study. To investigate the influence of scanner and field strength, we selected a few control participants with long follow-up times. These participants were scanned frequently because of a genetic disorder, thus cannot be considered truly healthy controls. However, they all have normal brain MRI and development, and we therefore considered them as examples of typical brain and skull development, allowing us to look into effects of scanner. In these participants, we did observe some systematic effects of scanner, but these were limited and considerably smaller than disease-related effects in the MLD and MS cohorts. In addition, a hardware upgrade after baseline for the pwMS and age-matched controls may also have affected subtle longitudinal changes. However, as this upgrade affected both groups, any resulting bias is unlikely to explain the observed differences between these two groups. In particular, since no corresponding changes were seen in the control group, the effects in pwMS were larger than any upgrade-related effects. Finally, the consistency of our findings across multiple scanners may be considered a strength, as it underscores the robustness of the observed disease-related effects. For future applications, it would be worthwhile to evaluate whether image or post-processing harmonization techniques can mitigate scanner effects in the assessment of skull thickness and ICV (Zuo et al., 2023, Reynolds et al., 2023). Another limitation of this study is the accuracy of skull thickness measurements when T2w images with full brain coverage were not available. For quite some participants with only T1w images, we had to discard skull thickness measurements due to inaccurate segmentations. In addition, it should be noted that both ICV and skull thickness are MRI-derived measures. Skull thickness may be influenced by the choice of input images (as shown in the current paper, regarding the use of T1 only or T1 and T2 images), and ICV may be influenced by the choice of segmentation methods, which may yield different results (Nerland et al., 2022). Finally, we did not analyse MS subtypes in this study, which may have distinct effects on ICV and skull thickness.

In conclusion, this study provides evidence that in adult individuals with MLD and MS, the skull thickness increases and ICV decreases. We further show that ICV and skull thickness are inversely associated. Finally, analyses of skull sub-regions suggest that the observed changes are diffuse, rather than localized to a specific region. These structural changes may influence volumetric analyses, as ICV shrinkage could lead to underestimation of atrophy when normalized volumes are used.. A similar phenomenon may occur in other neurodegenerative disorders, but this is currently unknown. Further research is needed to explore the underlying mechanisms and their potential impact on volumetric outcomes to allow more accurate evaluations of longitudinal brain atrophy progression.

## CRediT authorship contribution statement

**Guus H.J. Vorst:** Writing – review & editing, Writing – original draft, Visualization, Software, Methodology, Investigation, Formal analysis, Data curation, Conceptualization. **Nicole I. Wolf:** Writing – review & editing, Investigation, Data curation, Conceptualization. **David R. van Nederpelt:** Writing – review & editing, Validation, Software, Methodology, Formal analysis, Data curation. **Frederik Barkhof:** Writing – review & editing. **Marjo S. van der Knaap:** Writing – review & editing, Data curation. **Menno M. Schoonheim:** Writing – review & editing, Data curation. **Eva M.M. Strijbis:** Writing – review & editing, Data curation. **Petra J.W. Pouwels:** Writing – review & editing, Visualization, Validation, Software, Methodology, Investigation, Formal analysis, Data curation, Conceptualization.

## Funding

This research did not receive any specific grant from funding agencies in the public, commercial, or not-for-profit sectors.

## Declaration of competing interest

The authors declare the following financial interests/personal relationships which may be considered as potential competing interests: The authors of this manuscript declare relationships with the following companies: N.I. Wolf is the initiator of the deferiprone trial for Pelizaeus-Merzbacher disease and advisor and/or local PI for premarketing clinical trials in Metachromatic Leukodystrophy and other leukodystrophies (Shire/Takeda, Ionis, PassageBio, VigilNeuro, Sana Biotech, Lilly, Calico), consulting fees are paid to the institution. She is member of the MLD initiative and the European Reference Network “Rare Neurological Diseases” (ERN-RND), project ID 739510. D.R. van Nederpelt is supported by the European Union’s Horizon Widera programme under grant agreement no. 101159624 (TACTIX). F. Barkhof is part of the Steering committee or Data Safety Monitoring Board member for Biogen, Merck, Eisai and Prothena. Advisory board member for Combinostics, Scottish Brain Sciences, Alzheimer Europe. Consultant for Roche, Celltrion, Rewind Therapeutics, Merck, Bracco. Research agreements with ADDI, Merck, Biogen, GE Healthcare, Roche. Co-founder and shareholder of Queen Square Analytics LTD. M.M. Schoonheim serves on the editorial board of Neurology, Multiple Sclerosis Journal and Frontiers in Neurology, receives research support from the Dutch MS Research Foundation, Eurostars-EUREKA, ARSEP, Amsterdam Neuroscience and ZonMW (Vidi grant, project number 09150172010056) and has served as a consultant for or received research support from Atara Biotherapeutics, Biogen, Celgene/Bristol Meyers Squibb, EIP, Sanofi, MedDay and Merck. E.M.M. Strijbis serves on the editorial board of Neurology and Frontiers in Neurology and has received speaker fees from Merck and Novartis. Given their role as editorial board members, M.M. Schoonheim and E.M.M Strijbis had no involvement in the peer review of this article and had no access to information regarding its peer review. Full responsibility for the editorial process for this article was delegated to another journal editor All other authors report no known competing financial interests or personal relationships that could have appeared to influence the work reported in this paper.

## Data Availability

Data will be made available on request.
