# Supplementary material for: Longitudinal MRI-based changes in intracranial volume and skull thickness observed in both metachromatic leukodystrophy and multiple sclerosis
Source: Neuroimage Clin. 2026 Feb 16;49:103968. doi: 10.1016/j.nicl.2026.103968 (PMC12934227; doi:10.1016/j.nicl.2026.103968)
Supplement: Supplementary Data 1 [file mmc1.docx]

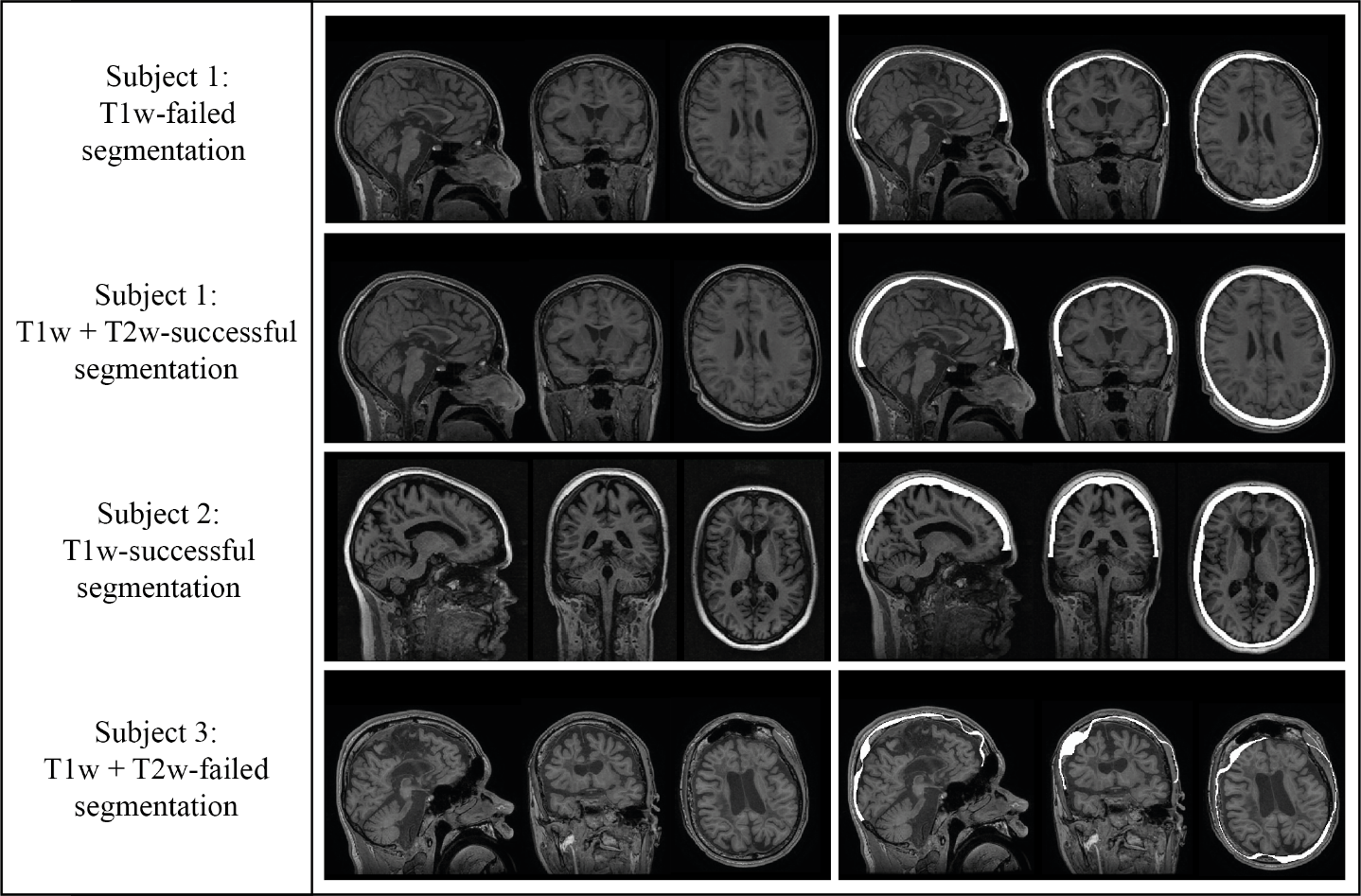


**Supplementary Figure 1. Example quality control of skull segmentation through BETsurf from differential input.** From top to bottom, rows show three representative MLD subjects segmented under 4 conditions: (i) Subject 1: T1w scan with a failed segmentation, (ii) Subject 1: T1w+T2w scan with successful segmentation, (iii) Subject 2: T1w scan with successful segmentation, and (iv) Subject 3: T1w+T2w scan with failed segmentation. For each row, the left panel displays the original T1 scan, and the right panel shows the same scan with the skull segmentation mask overlaid. Both panels display sagittal, coronal and axial views. These examples illustrate both successful and failed cases.


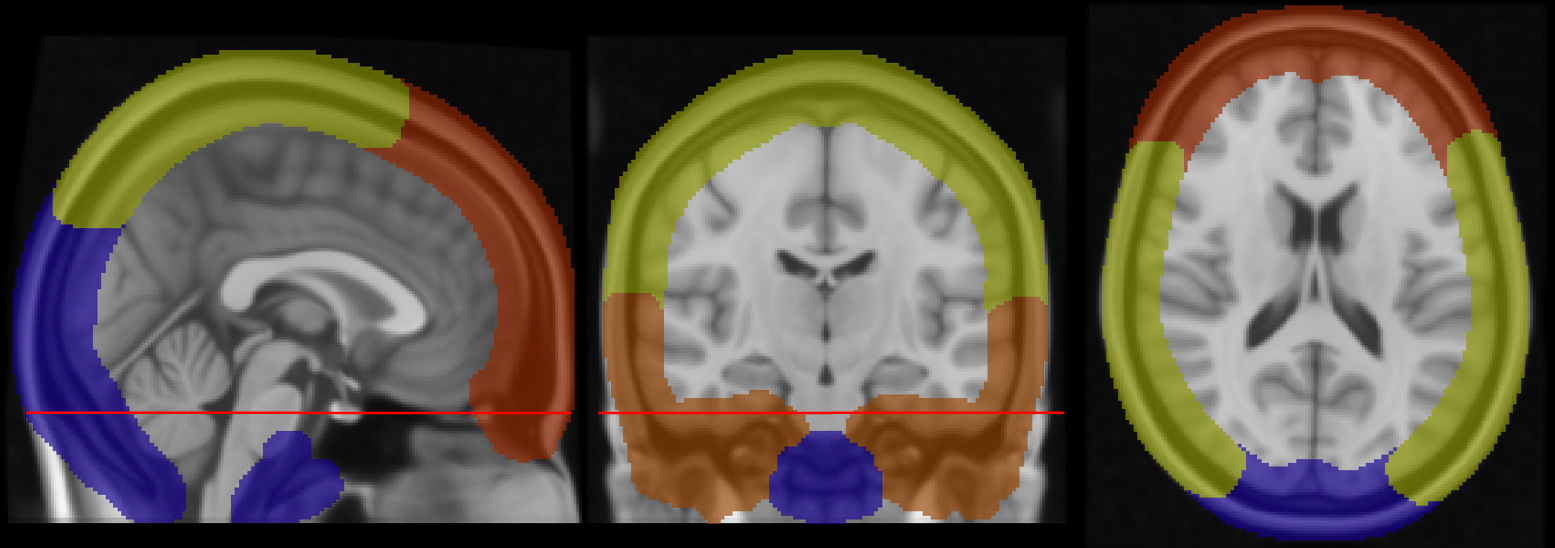


**Supplementary Figure 2.**  **The regional segmentation mask of the 4 skull regions**. Frontal (red), parietal (yellow), occipital (blue), and temporal (orange) in MNI-2mm space after being dilated with an 8mm Gaussian kernel. The red line indicates the z=28 cut- off limit, skull below this line was excluded.


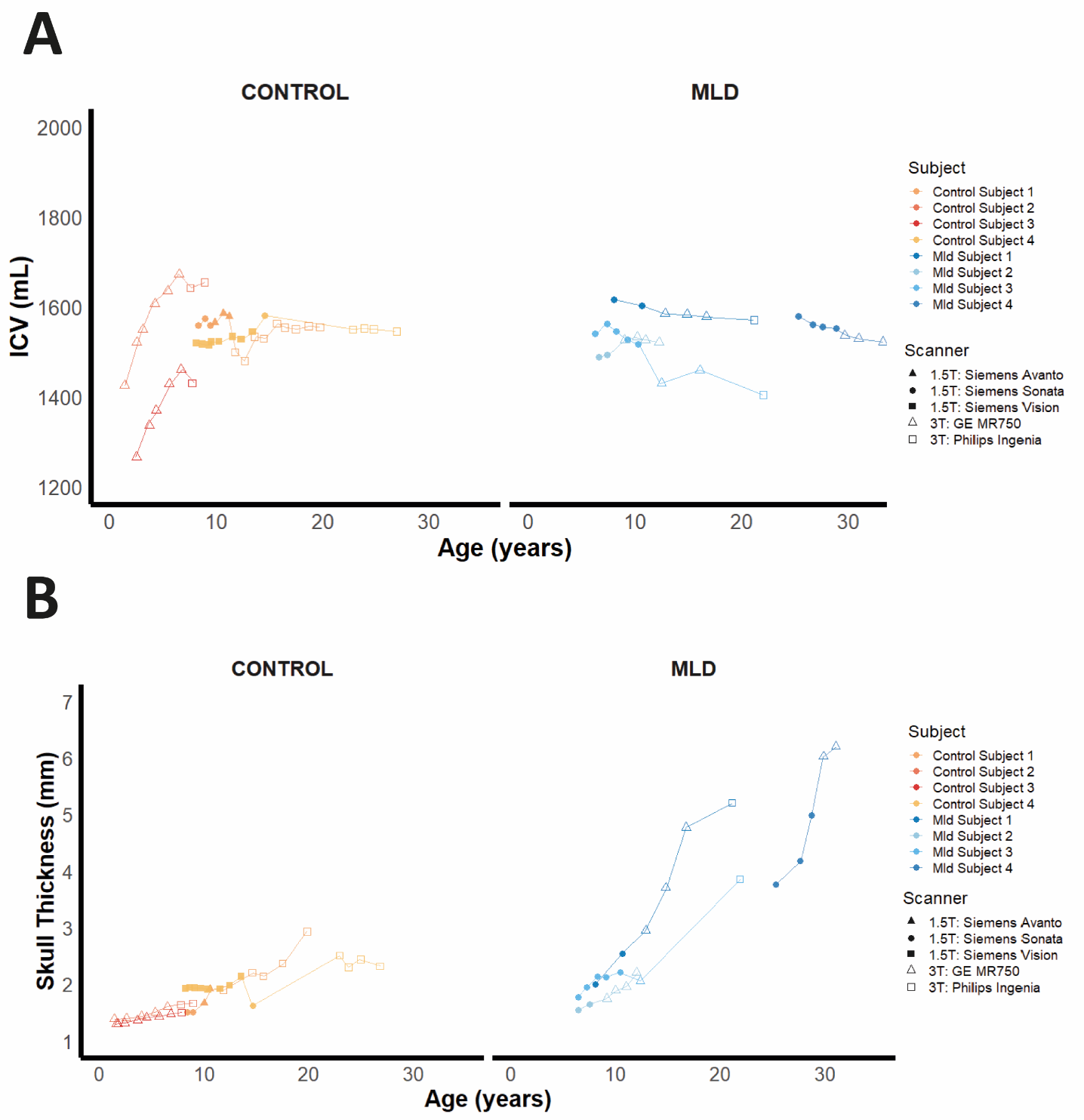


**Supplementary Figure 3. Longitudinal changes in ICV and skull thickness over time by scanner type.** Data is represented as (a) ICV and (b) skull thickness, as a function of age in 4 controls (left) and 4 MLD subjects (right), scanned on multiple scanners. Each colour represents an individual subject. Each point represents an individual measurements. Each shape represents a different scanner. Y-axes and scaling for ICV and skull thickness are identical to those used in Figure 2 to allow for direct visual comparison.

**B**

**A**


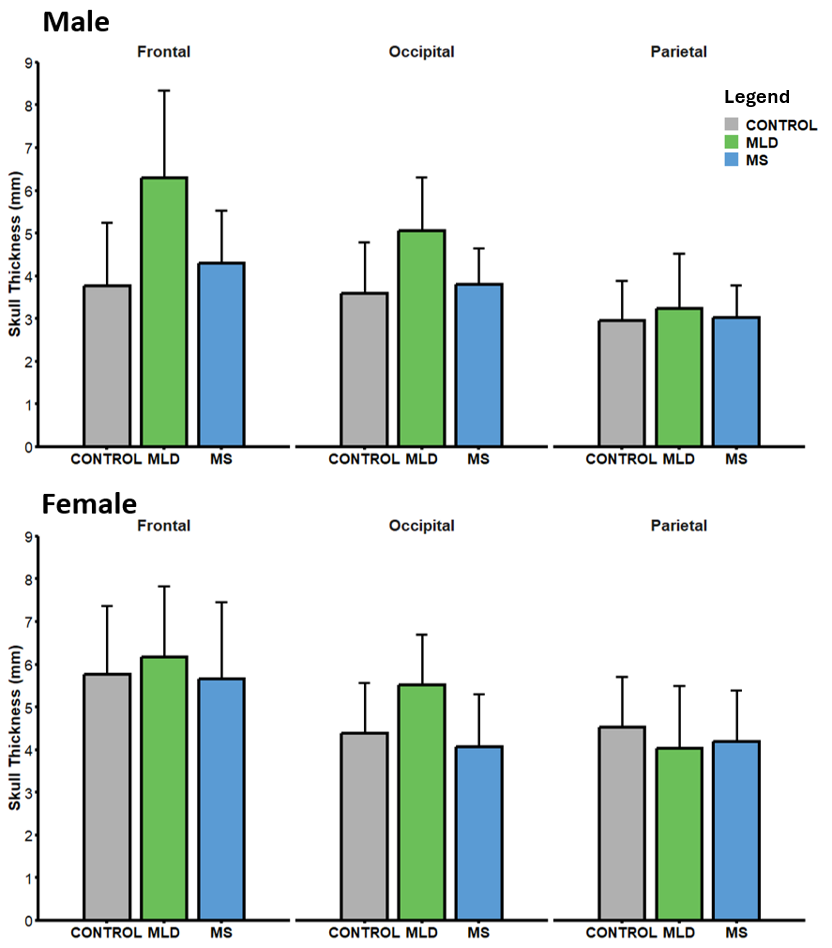


**Supplementary Figure 4. Bar plots of regional skull thickness of the frontal, occipital and parietal skull regions.** Data is represented in bars for Males (upper) and Females (lower) separately, including only measurements above 20 years. Each bar represents the mean skull thickness (in mm), with error bars indicating the standard deviation based on the average skull thickness per dataset for each skull region. Colours indicate Control (grey), MLD, (green), and MS (blue).
